# Diphtheritic Croup

**Published:** 1881-10-20

**Authors:** J. M. Batten

**Affiliations:** Pittsburg, Penn.


### DIPHTHERITIC CROUP.

BY J. M. BATTEN, M. D., OF PITTSBURG, PA.

Pseudo-membranous, membranous or diphtheritic croup has been, from time immemorial, considered a very grave and fatal disease. It is my opinion that the two diseases, membranous and diphtheritic croup, are distinct diseases, and require, in many cases, distinct treatment. It is possible to confound the one disease with the other; for, on account of the great fatality of the disease, we are not often offered an opportunity of studying their sequelæ.

Diphtheritic croup is a constitutional disease with local symptoms and sequelæ of albuminuria, paralysis, debility and perhaps heart paralysis. Membranous croup is a hyper-fibrination of the blood; children teething, with a large amount of fibrin in the blood, are likely to be attacked with membranous croup; besides, the latter disease has no sequelæ. I will not, however, stop to discuss the pathology of the disease, but will pass on to give you the history and treatment of a case of diphtheritic croup.

The case I am about to relate is a good type of the disease, and presented all the symptoms which might indicate a fatal termination.

I saw James H. W., aged 6 years, on November 2d, 1880. He had been sick about a week, with what the mother stated was a bad cold. Upon examination, the throat was red, with a patch of membrane behind each tonsil; the tonsils were not very much enlarged, the sub-maxilary glands were slightly enlarged, the tongue was coated, the breathing was labored and abnormal; there was aphonia; the cough was dry and had a ringing, whistling sound; the patient had been restless the previous night. I immediately imparted my opinion to the family that the case would likely terminate fatally. I put the child on No. 1:

℞ Bromid. potass.................................. gr. l,

Chlorat. potas.................................. gr. l,

Tinct. aconiti rad.............................. gtt. xij,

Syr. tolutani.................................. } aa ʒij,

Syr. senegæ.................................... }

Aquæ............................................ ℥ss.

M. Sig. A teaspoonful every three hours.

The following day the symptoms, if anything, were more grave; the child had perspired freely after having been placed in bed, in a warm, comfortable room. The eyes were glassy and ejected, breathing difficult, head thrown back. Countenance anxious, pulse 110 to 120. Respirations frequent; a dry mucous rale in both bronchia. The case was not one favorable for tracheotomy, for the membrane ramified the trachea and bronchia and bronchial tubes of both lungs. I continued the treatment till evening, with the addition of inhalation of lime steam, or steam from lime, when I put the patient upon No. 2:

| | | |
|---|---|---|
| R | Chloratis potass | gr. lxxx, |
| | Tinct. ferri chlor | gtt. clx, |
| | Syr. simplicis | ℥ss, |
| | Aquæ | ℥iss. |

M. Sig. A teaspoonful every three hours, and withdrew the first prescription.

I kept the patient on this until Friday morning, November 5th, with inhalation of steam from lime. There was no improvement; the father stated to me that the patient could not swallow the medicine during the previous night. I then put him on No. 3:

| | | |
|---|---|---|
| R | Hydrarg. chlor. mit | gr. iv, |
| | Pulv. ipecac | gr. ij, |
| | Albi sacch | gr. xx. |

M. Ft. chart. viij. Sig. One powder every three hours.

I told the father to give the second prescription as often as possible.

In the afternoon the patient commenced expectorating a thick, tenacious, muco-purulent matter, a half pint in quantity; after this the patient commenced to breathe easily, the cough became hoarse, expectoration easy, and he went to sleep for the first time since the night of November 1st (Monday night), The patient is now rapidly convalescing; has not regained his voice. During the little patient's sickness I gave whisky and nourishing diet freely. On examining the urine, November 7th, I found it loaded with albumen.

I continued the treatment with prescriptions No. 2 and No. 3, until November 8th, when I dropped No. 3, the calomel, and continued No. 2, tinct. chlor. of iron, etc.

Now, the question may be asked, was it prescription No. 2 or No. 3 that benefitted the patient. I believe it was prescription No. 2, together with the inhalation of steam from lime; but the calomel, if it did no good, did no harm, and took the place of prescription No. 2 when the child could not swallow. Now, this was an interesting case, and is the fifth patient with this disease, diphtheritic croup, I have seen, all of which died except the latter; and if no sequelæ should arise to endanger his life, I think my patient will convalesce rapidly. Throughout the disease my little patient was very restless, gasping for breath, and wanted to be fanned.—*Med. and Surg. Rep.*

